# Coronary Orbital Atherectomy Through Newly Deployed Left Main Coronary Stent

**DOI:** 10.7759/cureus.42821

**Published:** 2023-08-01

**Authors:** Tabitha N Lobo, Steven Ajluni, Akhil Mogalapalli, Sundeep Kumar, Tarek Hammad, Elsayed Abo-Salem

**Affiliations:** 1 Department of Internal Medicine, University Hospitals Cleveland Medical Center, Cleveland, USA; 2 Department of Cardiology, University Hospitals Cleveland Medical Center, Cleveland, USA; 3 Department of Cardiology, Saint Louis University Hospital, St. Louis, USA

**Keywords:** cto: chronic total occlusion, complex pci, percutaneous coronary intervention, coronary artery disease, orbital atherectomy

## Abstract

Percutaneous coronary intervention (PCI) in complex, calcified coronary lesions can be assisted with orbital atherectomy (OA). OA is generally avoided when there are lesions amendable to OA distal to a newly deployed stent due to the risk of device-stent interaction, burr entrapment, and stent avulsion. We present a case documenting the successful passage of an OA system through a recently deployed left main stent to prepare a chronically occluded left anterior descending for PCI.

## Introduction

Orbital atherectomy (OA) is approved by the Food and Drug Administration (FDA) for the treatment of calcified de novo coronary lesions in preparation for stent deployment [1,2]. When a complex percutaneous coronary intervention (PCI) involves a staged procedure, traversing a recently deployed stent may be necessary to intervene in significant distal coronary disease. There is a dearth of published articles regarding atherectomy through a recent stent due to risks of burr entrapment and stent avulsion [2-4]. Herein, we report a case that demonstrates the safe passage of the orbital atherectomy system (OAS) through a newly deployed left main (LM) coronary stent to perform a PCI on a chronic total occlusion (CTO) lesion of the left anterior descending (LAD) in a patient with multivessel disease, not eligible for surgical intervention. To the best of our knowledge, this is the first case report documenting the successful passage of the OAS through a recently deployed LM stent.

This article was previously presented as a meeting abstract at the 2021 American College of Cardiology (ACC) 70th Annual Scientific Session.

## Case presentation

A 72-year-old male with a prior history of essential hypertension, hyperlipidemia, and chronic kidney disease presented with non-ST-elevation myocardial infarction and cardiogenic shock. A coronary angiogram showed severe distal LM stenosis extending into the left circumflex artery (LCx) and ramus intermedius (RI) bifurcation accompanied by CTO of the proximal LAD. The patient underwent Impella CP (Abiomed, Danvers, Massachusetts) placement for hemodynamic support and successful bifurcation PCI of LM into LCx and RI. The attempt at LAD PCI was aborted due to severe calcification. After a multidisciplinary discussion, it was deemed that the patient was a suitable candidate for a staged complex PCI of the proximal CTO of LAD to achieve complete revascularization.

**Figure 1:**
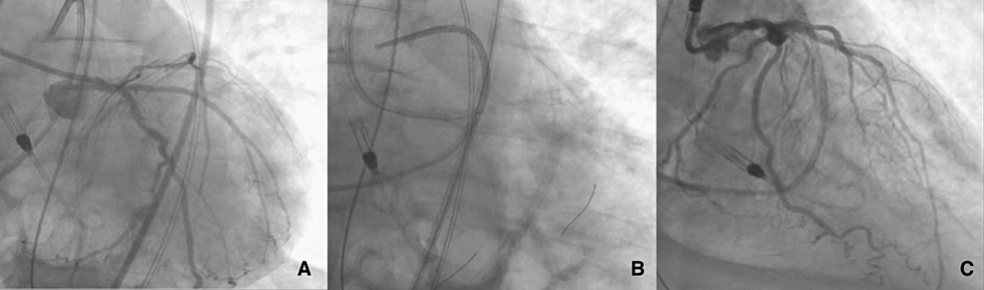
A: Pre-PCI coronary angiography. B: Coronary wire in LAD and LCx during LAD intervention. C: Post-PCI angiography showing patent LAD with good TIMI 3 flow in distal vessel bed demonstrating successful OA-guided percutaneous intervention of LAD artery OA: orbital atherectomy; LAD: left anterior descending artery; LCx: left circumflex artery; PCI: percutaneous coronary intervention; TIMI: thrombolysis in myocardial infarction.

The proximal CTO of the LAD was crossed using a Fielder XT wire (Asahi Intecc Medical, Aichi, Japan) that was advanced into the distal LAD. Balloon angioplasty was done using a 4.5 x 8 mm non-compliant balloon in the recently placed LM stent to achieve proximal optimization and full expansion in LM. We then exchanged the Fielder XT wire for a Viper Wire (CSI, St. Paul, Minnesota) over microcatheter which was advanced into LAD and a kissing 3.0 mm balloon was performed to open the recently placed LM stent struts. A 1.25 mm Diamondback 360 Coronary OA (CSI, St. Paul, Minnesota) was advanced into the LAD using glide-assist low speed (5k RPM) and then four runs were performed at 80,000 rpm followed by a single run at 120,000 rpm. A 3.0 x 33 mm drug-eluting stent was then deployed in proximal LAD using the TAP technique with a final kissing balloon (Figure 1). Intravascular ultrasound (IVUS) confirmed stent apposition and expansion in LAD and intact LM stent. Final angiography revealed no procedural complication. Impella CP was removed the next day and the patient was subsequently discharged a few days later in stable condition.

## Discussion

Advances in interventional coronary techniques have increased the complexity of lesions deemed to be candidates for PCI. There have been advances in percutaneous coronary management such that better revascularization techniques for highly calcified lesions and CTOs exist compared to previous methods [5].

OA is designed for calcified coronary plaques to optimize calcified lesion preparation and ensure maximal stent expansion [6]. The success of OA in calcified coronary lesions has allowed for expanded use in selected cases with in-stent restenosis (ISR), as reported in case studies [2,7]. However, having not been approved by the FDA, this off-label use must be practiced with caution to avoid deleterious stent interaction [3].

In a previously documented case report, PCI was performed through an existing stent. In this case, the OAS was used to intervene in severe stenosis in the LCx jailed by an LM-LAD stent that was placed two years prior. The procedure documented complications that included catheter entrapment and stent avulsion [8]. A literature search did not reveal any further cases in which OAS was used through a fresh stent. This is significant because anytime the OAS is passed through a stent there are associated complications that include device entrapment, stent distortion, stent fracture or dislodgement, burr entrapment, and coronary perforation [2,7,9]. In our case, proper guide engagement, proximal stent optimization, and maximum dilation of stent struts prior to atherectomy were keys to minimizing contact between bur and fresh stent. We used glide assist during advancement that helped with smooth crossing through an angle from LM into LAD. Table 1 represents reports in which OAS has been utilized for ISR and some complications documented. The use of IVUS and careful maneuvering of the device around the stent struts are some ways described to mitigate stent/device interactions and thus complications.

**Table 1:** Use of OA for ISR and complications reported OA: orbital atherectomy; ISR: in-stent restenosis; OAS: orbital atherectomy system.

Authors	Journal	Total patients	Peri-procedural complications
Saroj Neupane et al. [3]	Catheter Cardiovasc Interv. 2021	41	Two cases: Contained coronary perforations. Two cases: No-reflow myocardial infarctions. One case: Peripheral OAS used with subsequent stent strut damage
Keisuke Yasumura et al. [4]	Catheter Cardiovasc Interv. 2021	87	One case: Device/stent interaction with distal guide wire fracture and retained fragment
Yutaka Matsuhiro et al. [8]	JACC Case Rep. 2020	1	The catheter was entrapped within the stent and the stent was avulsed during catheter withdrawal
K Shaikh et al. [2]	Case Rep Cardiol. 2016	1	During withdrawal, device/stent interaction caused the orbital head of OAS to be enveloped by wire severed from the braided driveline

## Conclusions

Highly calcified lesions and CTOs have been associated with poor clinical outcomes, and PCI can be challenging in these cases. Advances in interventional coronary techniques have increased the complexity of lesions deemed to be candidates for PCI. In patients with highly calcified multivessel coronary disease that are poor surgical candidates and have characteristics that make them candidates for complex revascularization, the use of OA to prepare a lesion for PCI through a fresh stent is a feasible option in appropriately selected patients. Careful patient selection, appropriate preparation with adequate dilatation of the stent, and cautious advancement of the OAS with proper technique are essential for successful outcomes.
